# Wearable Devices in Health Monitoring from the Environmental towards Multiple Domains: A Survey

**DOI:** 10.3390/s21062130

**Published:** 2021-03-18

**Authors:** Mostafa Haghi, Saeed Danyali, Sina Ayasseh, Ju Wang, Rahmat Aazami, Thomas M. Deserno

**Affiliations:** 1 Peter L. Reichertz Institute for Medical Informatics of TU Braunschweig and Hannover Medical School, Braunschweig, 38106 Lower Saxony, Germany; ju.wang@plri.de (J.W.); thomas.deserno@plri.de (T.M.D.); 2 Faculty of Engineering, Ilam University, Ilam 69315-516, Iran; s.danyali@ilam.ac.ir (S.D.); sina.ayasseh@gmail.com (S.A.); r.aazami@ilam.ac.ir (R.A.)

**Keywords:** wearable devices, health monitoring, environmental domain, proactive medicine, personalized healthcare, IoMT, prediction and prevention

## Abstract

The World Health Organization (WHO) recognizes the environmental, behavioral, physiological, and psychological domains that impact adversely human health, well-being, and quality of life (QoL) in general. The environmental domain has significant interaction with the others. With respect to proactive and personalized medicine and the Internet of medical things (IoMT), wearables are most important for continuous health monitoring. In this work, we analyze wearables in healthcare from a perspective of innovation by categorizing them according to the four domains. Furthermore, we consider the mode of wearability, costs, and prolonged monitoring. We identify features and investigate the wearable devices in the terms of sampling rate, resolution, data usage (propagation), and data transmission. We also investigate applications of wearable devices. Web of Science, Scopus, PubMed, IEEE Xplore, and ACM Library delivered wearables that we require to monitor at least one environmental parameter, e.g., a pollutant. According to the number of domains, from which the wearables record data, we identify groups: G1, environmental parameters only; G2, environmental and behavioral parameters; G3, environmental, behavioral, and physiological parameters; and G4 parameters from all domains. In total, we included 53 devices of which 35, 9, 9, and 0 belong to G1, G2, G3, and G4, respectively. Furthermore, 32, 11, 7, and 5 wearables are applied in general health and well-being monitoring, specific diagnostics, disease management, and non-medical. We further propose customized and quantified output for future wearables from both, the perspectives of users, as well as physicians. Our study shows a shift of wearable devices towards disease management and particular applications. It also indicates the significant role of wearables in proactive healthcare, having capability of creating big data and linking to external healthcare systems for real-time monitoring and care delivery at the point of perception.

## 1. Introduction

For the year 2016, the World Health Organization (WHO) estimates that outdoor air pollution caused death of 4.2 million people worldwide and that 91% of the world’s population breath unhealthy air [1]. In particular, the WHO estimates that about 58%, 18%, and 6% of deaths in heart disease and stroke, in chronic obstructive pulmonary disease (COPD) and lung infections, and in lung cancer occurred due to air pollution, respectively [1]. In addition, WHO also estimates that 3.8 million people worldwide lose their life due to indoor air pollutants caused by cooking with stoves running on solid or biomass fuels or kerosene. Of these 3.8 million annual deaths, 27% were due to pneumonia, 27% due to heart disease, 18% due to stroke, 20% due to COPD, and 8% due to lung cancer who are vulnerable to air pollutants [2]. These statistics indicate the significant and intensive impact of environmental factors on human health.

Air pollutants are toxic and hazardous gases such as carbon dioxide (CO${}_{2}$), volatile organic compounds (VoCs), nitric oxide (NO), nitric dioxide (NO${}_{2}$), particular matter (PM), ozone (O${}_{3}$), carbon monoxide (CO), sulfur dioxide (SO${}_{2}$), methane (CH${}_{4}$), ammonia (NH${}_{3}$), teledyne (C${}_{7}$H${}_{4}$), hydrogen peroxide (H${}_{2}$O${}_{2}$), and ethanol (C${}_{2}$H${}_{5}$OH). Other environmental factors that have an impact on health and wellbeing are, for instance, the amount of ultraviolet (UV) radiation, the light intensity and daylight length, the sound level (SL), and air conditions such as temperature (T), humidity (H), and pressure (P) [3,4,5]. In particular, UV radiation is proven as major cause of skin cancer [6,7]. A high SL has detrimental effects on sleep quality and the nervous system. Specifically, these effects are higher in patients suffering from chronic diseases, such as heart disease and hypertension [8,9].

However, factors influencing health are not restricted to the environmental domain. The WHO also recognizes behavioral, physiological, and psychological domains relevant for quality of life (QoL) and health, impacting subjects with chronic diseases and elderly people [10]. Furthermore, factors of the environmental domain affect the other domains. [11]. Mobile health (mHealth) [12], electronic health (eHealth) [13], and the Internet of medical things (IoMT) [14] shift the traditional methodology (i.e., symptom → diagnosis → treatment) towards health protection (i.e., monitoring → prediction → prevention) [15]. This includes continuous, comprehensive, and simultaneous monitoring of all important influencing domains effecting healthcare [16]. At present, the management of chronic diseases is mostly reactive, which imposes additional costs and efforts on patient care systems [17]. The new healthcare model is predictive, preventive, personalized, and participatory (4P) [18]. Unlike the current reactive treatment, it proactively focuses on the causes of diseases rather than its symptoms. Proactive healthcare may indicate symptoms of diseases or disorders long before their onset. Therefore, humans are motivated to evaluate (and, therefore, monitor) their lifestyle or health status, and to improve their QoL [17,19,20]. Applying proactive healthcare is economic, saves time, identifies chronic diseases at earlier stages, and increases the quality of service to patients [21]. Wearable devices are capable of monitoring environmental, behavioral, physiological, and psychological parameters [22]. The wearables can measure and interchange heterogeneous data from different domains, based on wireless body area network (WBAN) [23]. Using wireless sensor network (WSN) and WBAN approaches, more sensors are integrated which increases efficiency and flexibility of data collection, processing, transmission, and analysis [24,25]. Recent advances in semiconductor technology, such as shrinking the sensor’s size or diminishing its power consumption, and information and communication technology (ICT) have increased the use of wearable devices in health monitoring [26,27,28], as well as have proven a variety of benefits [29,30,31]. Furthermore, increased convenience and less interference with daily activities foster continuous rather than partial monitoring. This eases medical decision-making [32].

In this work, we consider wearable devices for simultaneous monitoring of parameters from all four domains, emphasising on the environmental domain and its integration with three other domains. Based on a literature survey, we aim at:

- introducing criteria, principles, and features of wearable devices in healthcare and well-being,
- identifying groups of wearable devices regarding the domain(s) of monitoring (Gx) such that “G” and “x” indicate the group and the number of monitoring domains, respectively,
- identifying the applications of wearable devices,
- determining additional characteristics such as monitoring duration, costs, and convenience of use, data usage (propagation), and based on that:
- discussing the evolution of wearables, and
- proposing requirements for future wearables.

## 2. Materials and Methods

In this survey, we identify wearable devices for health monitoring in academia between 2009 and 2020 (April) from the databases Web of Science, Scopus, PubMed, IEEE Xplore, and ACM Library.

### 2.1. Selection Criteria

The International Data Company (IDC) defines five categories of wearables: watches, wristbands, clothing, ear wear, and others [33].Because of its direct and indirect impact to the other domains, we highlight the environmental domain, in particular, air pollutants. Accordingly, we include and exclude devices that are:

- Inclusion:
  - worn on body, including wrist, waist, arm, foot, and chest,
  - measuring at least one toxic/hazardous air pollutant,
  - monitoring zero to more parameters from the behavioral, physiological, or psychological domains,
  - systems of wearables using WBAN,
  - garments, boots, gloves, and helmets, and
  - providing multiple carrying modes (e.g., attachments to bicycles, backpacks, etc.), as well as wearability.
- Exclusion:
  - handheld, portable, or stationary, and
  - measuring behavioral, physiological, and/or psychological but not environmental parameters.

According to the WHO criteria, we differ and define four groups of wearables:

- *G1–Environmental*: devices measure at least a single toxic/hazardous air pollutant. The number of parameters by each device is not restricted and may include physical (UV, light, SL, and etc.), as well as air conditions (T, H, and P).
- *G2–Environmental and behavioral*: devices measure in the environmental and behavioral domains simultaneously: The G1 requirements apply and additionally, at least one single parameter from behavioral domain is recorded, e.g., physical activity. The domain includes parameters such as movement, gait, step counting, climbing, and body posture. For example, a wearable equipped with VoC and accelerometer is a G2 device.
- *G3–Environmental, behavioral, and physiological*: devices meet requirements G2 and measure at least one physiological parameter.The physiological domain includes parameters such as vital signs (e.g., heart rate, respiratory rate, blood pressure, and skin temperature) and non-vital signs (e.g., skin conductance). Therefore, a G3 device has to deliver a minimum three parameters from different domains.
- *G4–All domains*: devices measure parameters from environmental, behavioral, physiological, and psychological domains.Psychological domain includes the mood and emotion of a subject, measuring the parameters such galvanic skin response (GSR).

### 2.2. Methodology of Analysis

**Figure 1:**
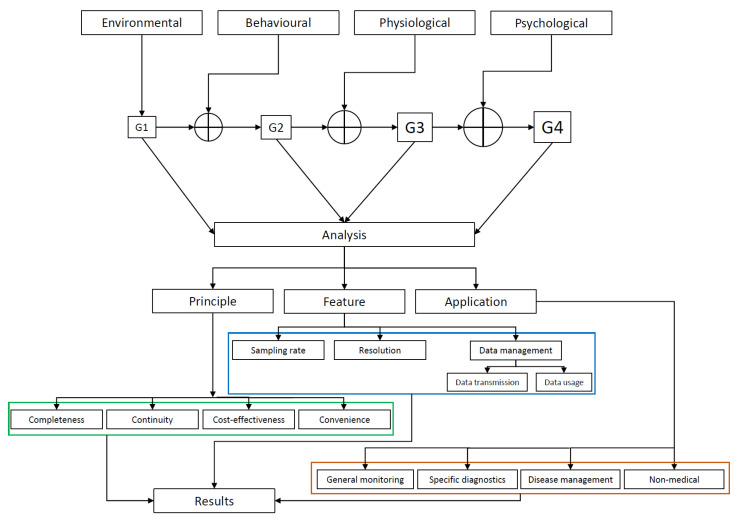
The methods of study and analysis.

We analyze the devices and deliver descriptive and comparative statistics according to four principles of group, continuous monitoring (duration/power and current consumption), cost, and mode of wearability, features and specifications, and applications (Figure 1).

#### 2.2.1. Principle

In previous work, we have introduced complete, continuous, cost-effective, and convenient (C4) monitoring as evaluation criteria for the performance of wearable devices [22]. Here, we apply these principles to specify each wearable and provide technical details, if available. The principles are:

- *Completeness*: recognizes the number of domains according to the WHO definition. Due to missing integration of the psychological domain, so far, none of the wearables are complete.
- *Continuity*: accomplishes the transition from only partial to ongoing patient monitoring. The period of monitoring is provided in hours or days, and information on power (mW) and current (mA) consumption support an estimate of battery run-time.
- *Cost-effectiveness*: is important for users to participate in proactive medicine. The embedded system design significantly impacts the cost of a prototype. If data is available, we compare to well-known products in market in terms of expense and bill of material (BoM).
- *Convenience*: addresses the mode of wearability. Dimension, compactness, and weight are important factors. In general, wrist-worn devices have a better convenience.

#### 2.2.2. Features

We investigate the performance and efficiency of wearables, considering sampling rates, resolution, data transmission (storage), and data usage (propagation). We present this information as provided by the authors of identified papers.

- *Sampling rate*: is the number of measurements per second (Hz) for a particular sensor. From the one side, increasing the sampling rate improves the real-time monitoring; but, from the other, it dissipates more power and storage and reduces the run-time. Hence, configuring a sensor’s sampling rate is a trade off and depends on the application.
- *Resolution*: the better the resolution the higher the reliability and effectiveness in data fusion and decision-making. We also consider the limit of detection (LoD) as the lower boundary, and the sensor’s coverage range.
- *Data management*: includes the data acquisition, storage, transmission, and propagation. Disease diagnostics and decision-making depends on the fusion of individual parameters and post-processing. Data management includes two steps:
  1. Data transmission: for fusing, devices have to transmit the data using short- and long-range protocols. We also consider devices that transmit data but store it locally (Table 1).
    – *Short-range (SR)*: it addresses the protocols such as Bluetooth, Bluetooth Low Energy (BLE), and Zigbee.
    – *Long-range (LR)*: it addresses the protocols such as long-range (LoRa), wireless local area network (WLAN) or wireless fidelity (WiFi). We also consider devices that transmit data but store it locally (Table 1).
  2. Data usage (propagation): depending on mode of data transmission and aim of measurement, the second step is implemented. Hence, four categories are identified:
    – *Internal memory*: The wearable does not transmit data but stores it on the device, which we consider as a passive node that is disconnected to external systems.
    – *Smartphone*: The wearable transmits data in short-range to a smartphone for monitoring, processing, and/or visualization. Such devices potentially can be integrated into healthcare system for bidirectional communication with medical personnel in an emergency.
    – *Computer*: The wearable transmits data in short- or long-range to a personal computer (PC), desktop or a similar device for further processing and analysis. We consider the computer as the base for real-time monitoring and alerting (e.g., a fire station).
    – *Cloud or server*: The wearable propagates data in three tiers. The gateway might be a smartphone or any other embedded system capable of data receiving and transmitting.

#### 2.2.3. Application

We further analyze the application of the device. We classify into four categories:

- *General monitoring*: are devices that only deliver data without any specific application. These devices neither target particular users nor send further notifications to their users.They usually are applied for user information without further processing or linkage to healthcare systems. Such devices do not support care delivery. Some examples are devices in air quality, air pollution, and environmental monitoring.
- *Specific diagnostics*: are devices that have been designed, tested, and validated for particular situations and conditions, e.g., identification of a drunk driver. We specify the applications or user groups, e.g., specific age or firefighters.
- *Disease management:* are devices that have been particularly tuned for patients suffering from a specific disease, such as diabetes, Alzheimer, respiratory diseases, COPD, or asthma.
- *Non-medical* are devices that have been designed and tested under realistic scenarios in a primarily non-medical context, e.g., safety for industrial workers.

## 3. Results

**Figure 2:**
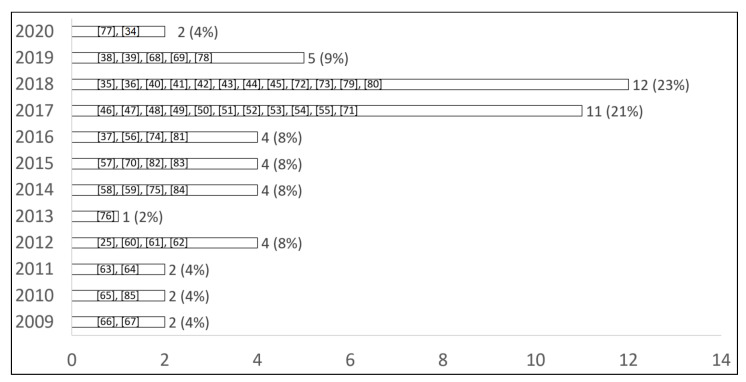
Total number of wearable devices that met the requirements of the study.

The total number of 53 papers published between 2009 and 2020 met the requirements of this study (Figure 2). Our analysis delivers 35 devices of G1, 9 devices of G2, and 9 devices of G3. G4 has not yet been reported on.

Temperature and humidity are monitored by 38 and 34 devices, respectively. Toxic gases such as CO${}_{2}$ and NO${}_{x}$ are measured by 14, 13, and 13 devices from G1, G2, and G3, respectively. VoCs, CO, and O${}_{3}$ are known as the hazardous gases with an adverse effect on health, each integrated into 12 devices. The list of environmental parameters is getting longer with particular matter, pressure, and SO${}_{2}$ by 13, 13, and 7 devices, respectively. The ambient physical parameters with less immediate danger to human health are at the bottom of the list: UV, light, and sound level are measured by 7, 5, and 5 devices, respectively.

**Figure 3:**
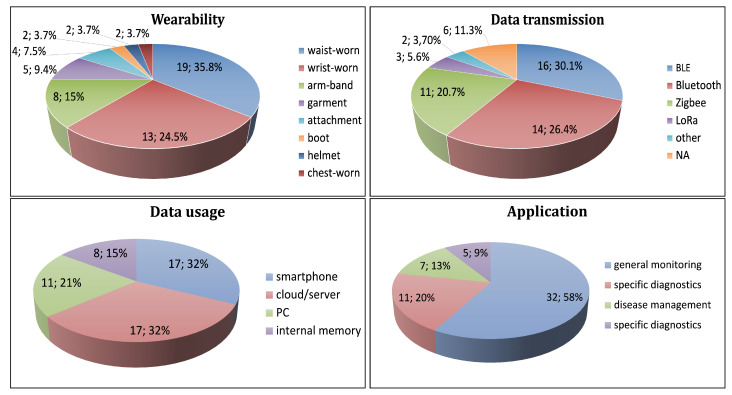
Distribution of wearable devices according to: mode of wearability (**top-left**), data transmission (**top-right**), data usage (**bottom-left**), and application (**bottom-right**) of the wearable devices.

Thirty-five and eight tenths percent of the devices are waist-worn (18 in all three groups). Wrist-worn and arm-worn are 24.5% (12) and 15% (8) of the devices, respectively. Nine and four tenths percent (5) of the devices are integrated into garment. We specify the remaining devices as other modes of wearability: 7.5% (4) attachments, 3.7% (2) boot, 3.7% (2) helmet, and 3.7% (2) chest-worn (Figure 3, top-left).

Usually, the measurement is followed by data transmission to a second party (gateway) for collection. Among all, BLE is the most frequently used protocol (30.1%) as it consumes less power than standard Bluetooth or Zigbee, which is used by 26.4% (14) and 20.7% (11) of the devices, respectively. Only 5.6% (3) of the devices transmit via LoRa, 6 devices do not specify the protocol, 1 device uses other protocols (WiFi), and 1 device stores the data on the internal memory (Figure 3, top-right).

Sixty-four percent (34) of the devices transmit the data to a smartphone or cloud/server, each with 32% (17). Twenty-one percent (11) of the devices transmit to a PC, and only 15% (8) of the devices store the data locally (Figure 3, bottom-left).

Fifty-eight percent (32) of the devices are used in general monitoring, and 20% (11) of the devices are applied in specific diagnostics. In these devices, the target subjects or/and the study have been specifically defined. Thirteen percent (7) of the devices are used in disease management, and 9% (5) of the devices are applied in non-medical (Figure 3, bottom-right).

### 3.1. G1–Environmental Monitoring

**Figure 4:**
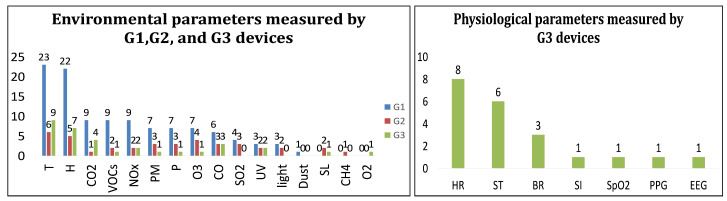
Measured parameters and iterations in G1, G2, and G3 (**left**) and physiological parameters measured by G3 (**right**) devices.

In total, 22 different environmental parameters are measured by G1 devices. Temperature and humidity are measured by 23 and 22 devices, respectively. Nine devices measure CO${}_{2}$, NO${}_{x}$, and VoCs, which indicates the importance of air pollutants in environmental monitoring. Particular matter, pressure, and O${}_{3}$ are, each integrated in 7 devices. CO and SO${}_{2}$ are integrated into 6 and 4 devices, respectively. UV and light are measured by 3 devices (Figure 4, left).

**Figure 5:**
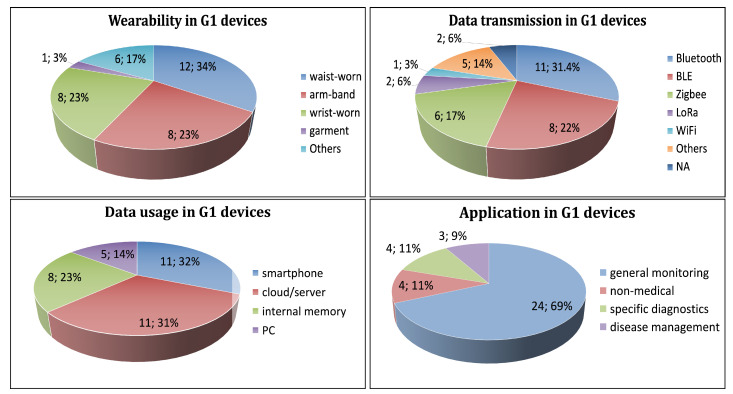
Distribution of G1 wearable devices according to: mode of wearability (**top-left**), data transmission (**top-right**), data usage (**bottom-left**), and application (**bottom-right**) of the wearable devices.

Eight percent of G1 wearables are waist-, wrist-, or arm-worn. Waist-based monitoring is the most popular mode of wearability with 34% (12), wrist- and arm-worn modes are 23% (8) of the devices. Garments and other types are 20% (7) devices (Figure 5, top-left).

Bluetooth is the most used protocol with 31% (11) of the devices, followed by BLE and Zigbee with 22% (8) and 17% (6) of the devices, respectively. LoRa is used in 2 devices. WiFi is utilized by only 1 device. The protocols of two devices are unspecified (Figure 5, top-right).

Thirty-two percent (11) of the devices each transmits the data to a smartphone and a cloud/server. Twenty-three percent (8) of the devices transmit to a PC. Only 14% (5) of the devices store data locally (Figure 5, bottom-left).

Sixty-nine percent (24) of the devices are used in general monitoring. Eleven percent (4) of the devices are applied in specific diagnostics. Eleven percent (4) of the devices are used in disease management, and 9% (3) of the devices are applied in non-medical (Figure 5, bottom-right).

We review the first group of wearable devices in terms of principles, features and specifications, and application, if available (Table 2 and Table 3).

### 3.2. G2–Environmental and Behavioral Monitoring

Measuring environmental and behavioral domains at the same time are considered in G2 wearable devices. In addition to capability of measuring physical activities by integrated accelerometer and IMU, G2 devices in this study measure 15 different environmental parameters yielding large diversity from air conditions and hazardous/toxic air pollutants to ambient physical elements.

Temperature, humidity, and O${}_{3}$ are measured by 6, 5, and 4 devices, respectively. Three devices measure CO, SO${}_{2}$, particle matter, and pressure which are the second popular environmental parameters. Three out of these 4 recent parameters are air pollutants which complies the essential needs of monitoring and the effect on human health. VoCs and NO${}_{2}$ as the air pollutants, as well as UV, light, and sound level, each, are integrated into 2 devices. CO${}_{2}$ and CH${}_{4}$ stand at the bottom and are measured by 1 device (Figure 4).

**Figure 6:**
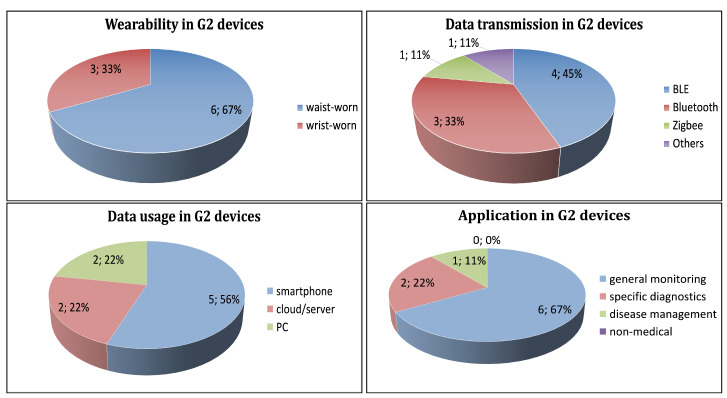
Distribution of G2 wearable devices according to: mode of wearability (**top-left**), data transmission (**top-right**), data usage (**bottom-left**), and application (**bottom-right**) of the wearable devices.

G2 wearable devices are waist- or wrist-worn. 67% (6) of the devices are waist-worn and 33% (3) of the devices are worn on wrist (Figure 6, top-left).

BLE is the most used protocol with 45% (4) of the devices, followed by Bluetooth standard with 33% (3) of the devices and Zigbee with 11% (1) of the devices, respectively. The protocol of 1 device is not specified (Figure 6, top-right).

Fifty-six percent (5) of the devices transmit the data to a smartphone. Twenty-two percent (2) of the devices transmit the data to a cloud/server. Twenty-five percent (2) of the devices transmit the data to a PC (Figure 6, bottom-left).

Sixty-seven percent (6), 22% (2), and 11% (1) of the devices are used in general monitoring, specific diagnostics, and disease management, respectively (Figure 6, bottom-right).

**Table 2:** Wearable devices in environmental monitoring (G1): part I.

Device	Wearability	Parameters	Energy Consumption	Data Transmission
Fresh Air [34]	wrist-worn	NO${}_{2}$, VoCs	NA	NA
[35]	wrist-worn	Formaldehyde, T, H	7 days	SR: BLE, ext. mem.
[36]	waist-worn	VoCs, T, H	39–52 h	Ext. mem.
W-Air [37]	wrist-worn	O${}_{3}$,CO${}_{2}$, T, H	50 mA, 5 h	SR: BLE
WE-Safe [38]	wrist-worn	CO${}_{2}$, CO, T, H, UV	33 mA	LR: LoRa
WE-Safe [39]	wrist-worn	CO${}_{2}$, T, H, UV	53.9 mA	LR: LoRa
[40]	helmet	CO${}_{2}$, CO, VoC, T, H, P, UV, light	NA	LR: WiFi
[41]	attachment	CH${}_{4}$, T	13 days	SR: Zigbee
[42]	arm-band	VoCs, T, H	NA	SR: Bluetooth
[43]	waist-worn	CO, SO${}_{2}$, NO${}_{2}$, O${}_{3}$, PM (1, 2.5, 10), T, H, P	NA	SR: BLE
[44]	waist-worn	CO, SO${}_{2}$, NO${}_{2}$, O${}_{3}$, NO, PM (2.5, 10), VoCs, T, H, P	NA	SR: BLE
[45]	waist-worn	PM (2.5), T	NA	SR: BLE
[46]	ring-worn	DNT, H${}_{2}$O${}_{2}$, MPO${}_{x}$	7.5 h	SR: BLE
[47]	waist-worn	PM (1, 2.5, 10), T, H	NA	SR: Zigbee
[48]	wrist-worn	CO${}_{2}$, T, H, P, light	64 mA	SR: Zigbee
[49]	attachment	CO${}_{2}$,T, H	5.07 days	SR: Zigbee
[50]	waist-worn	NH${}_{3}$, NO${}_{2}$, C${}_{7}$H${}_{8}$	NA	SR: BLE
[51]	wrist-worn	O${}_{3}$, T, H	NA	Ext. mem.
[52]	arm-band	ethanol (C${}_{2}$H${}_{5}$OH)	NA	SR: Bluetooth
[53]	waist-worn	O${}_{3}$, dust, T, H	NA	Ext. mem.
[54]	waist-worn or chest-worn	O${}_{3}$, PM (2.5, 10), T	150.96 mA	Ext. mem.
MyPart [55]	wrist-worn	PM (2.5, 10), T, H	75 mA	SR: BLE
[56]	arm-band	VoCs	NA	SR: Bluetooth
[57]	waist-worn	CO, NO${}_{2}$, SO${}_{2}$, T, H, P	18 mA, 65 h	SR: Bluetooth
[58]	arm-band	CO${}_{2}$, NO, PM	NA	SR: Bluetooth
[59]	waist-worn	CO, T, P	NA	SR: Zigbee
Citisense [60]	waist-worn	NO${}_{2}$, CO, O${}_{3}$, T, H, P	5days	SR: Bluetooth
SiNOxSense [61]	attachment	NO${}_{x}$	110.5 mA, 9 h	SR: Bluetooth
iEGAS [62]	waist-worn	SO${}_{2}$, T, H	NA	Ext. mem.
[26]	arm-band	VoCs, T, H	10 h	SR: Bluetooth
MAQS [63]	arm-band	CO${}_{2}$, T, H, light	5.5 to 24 h	SR: Bluetooth, ext. mem.
[64]	arm-band	total hydrocarbons, total acids, T, H	9 h	SR: Bluetooth
Wear-Air [65]	T-shirt	VoCs	NA	NA
[66]	boot	CO${}_{2}$	5 h	SR: Zigbee
[67]	arm-band	VoCs, T, H	NA	SR: Bluetooth

**Table 3:** Wearable devices in environmental monitoring (G1): part II.

Device	Sampling Rate (H)	Resolution/LoD/Coverage	Application	Data Usage
Fresh Air [34]	NA	>180 g/mol in working temp 4 °C to 23 °C	asthma management	internal memory
[35]	NA	30 ppb to 10 ppm	asthma management	cloud/server
[36]	10-min time interval	80 mL/min air flow, 300 ppb to 5 ppm	environmental	internal memory
W-Air [37]	2 Hz	VOC sensor: 0 to 1187 ppb	air pollution	cloud/server
WE-Safe [38]	NA	CO${}_{2}$: 0 ppm to 10,000 ppm	environmental for safety applications	cloud/server
WE-Safe [39]	NA	CO${}_{2}$: 0 to 10,000 ppm. T: −45 °C to +85 °C. H: 0 to 100%, UV: 1 to 11 index	environmental for safety applications	cloud/server
[40]	NA	CO: 0 ppm to 0.5 ppm, CO: 0 ppm to 2000 ppm, temp.: −40 °C to +85 °C, P: 300 hpa to 1100 hpa	environmental at micro climate scale	PC
[41]	NA	NA	monitoring environmental for workers in gas and oil industries	internal memory
[42]	350 mL\min	T: −45 °C to 125 °C. H: 0 to 100% RH	environmental air quality	cloud/server
[43]	NA	NA	air quality	smartphone
[44]	NA	NA	air quality	cloud/server
[45]	25 Hz	NA	air quality	smartphone
[46]	10 Hz	4 ppm in liquid phase	monitoring of explosive and nerve-agent threats in vapor and liquid phases	PC
[47]	0.5 Hz	NA	air quality	cloud/server
[48]	NA	CO${}_{2}$: 0 ppm to 10,000 ppm	environmental	cloud/server
[49]	NA	CO${}_{2}$: 0 ppm to 50,000 ppm, T: −40 °C to +124 °C, H: 0 to 100% RH	air quality in industrial environments	PC
[50]	350 mL\min(in test)	C7H8 (10 ppm exposure in test), NO2 & NH${}_{3}$ (1000 ppm exposure in test)	air pollution	smartphone
[51]	NA	NA	air quality	internal memory
[52]	NA	1 ppm, 1 to 200 ppm	testing drunk drivers	smartphone
[53]	NA	NA	air pollution	internal memory
[54]	NA	O${}_{3}$ 0 ppm to 20 ppm, temp.: 0 °C to 100 °C	respiratory disease	internal memory
MyPart [55]	NA	NA	air quality	smartphone
[56]	NA	1 ppm	air quality	smartphone
[57]	1 Hz	P: 50 KPa to 115 KPa. CO: 0 ppm to 13 ppm, SO${}_{2}$: 0 ppm to 10 ppm. NO${}_{2}$: 0 ppm to 27 ppm	air quality	smartphone
[58]	NA	NA	air quality	cloud/server
[59]	NA	temp.: −55 °C to +150 °C	industries and coal mines & homes	PC
Citisense [60]	NA	NA	air quality	cloud/server
SiNOxSense [61]	NA	NA	air quality	smartphone
iEGAS [62]	NA	NA	environmental	internal memory
[26]	1 Hz	4 ppb to 1000 ppm, T: 5 °C to 46 °C, H: 0 to 100% RH	environmental	smartphone
MAQS [63]	NA	NA	indoor air quality	cloud/server
[64]	NA	1 ppb, Max. T: 42.2 °C, H: 0 to 100% RH	environmental	smartphone
Wear-Air [65]	NA	NA	air quality & environmental	internal memory
[66]	NA	CO${}_{2}$: 0 ppm to 42,800 ppm	environmental in hazardous works	PC
[67]	NA	1 ppb, max. T: 23.8 °C, max. H: 100% RH	environmental	smartphone

In addition, only some of the developers have reported the final cost of the prototypes, which encompasses fabrication, components, and layout design. References [68,69,70,71] have reported 150$, 130$, 150$, and 140$ as the final cost of the prototypes, respectively.

We address principles, features and specifications, and application of G2 wearable devices in Table 4 and Table 5, if available.

**Table 4:** Wearable devices for simultaneous monitoring of environmental and behavioral domains (G2): part I.

Device	Wearability	Parameters	Energy Consumption	Data Transmission
ART [68]	wrist-worn	O${}_{3}$, TVoC, T, H, acceleration	18 h to 48 h	SR: BLE, ext. mem.
MLMS-EMGN-5.1 [69]	wrist-worn	SO${}_{2}$, NO${}_{2}$, CO, T, H, motion tracking, P, SL	28 h,15.8 mA	SR: BLE, ext. mem.
[72]	waist-worn	CO${}_{2}$, T, H, P, motion tracking, altitude	300 mW	SR: Bluetooth
AMAS [73]	waist-worn	PM${}_{2.5}$, oxidative potential(OP), light, acceleration	14 h	SR: Bluetooth, ext. mem.
[74]	waist-worn	CO, CH${}_{4}$, acceleration	1.6 W	SR: Zigbee
UPAS [70]	waist-worn	PM${}_{2.5}$, T, H, P, UV, acceleration	25 h to 43 h	SR: BLE, ext. mem.
[75]	wrist-worn	CO, NO${}_{2}$, O${}_{3}$,SO${}_{2}$, T, H, UV, motion tracking	3 days	SR: BLE, ext. mem.
Eco-Mini [76]	waist-worn	VoCs, O${}_{3}$, SO${}_{2}$, T, H, SL, light, acceleration	6 h to 12 days	SR: Bluetooth, ext. mem.
[71]	waist-worn	O${}_{3}$, PM, acceleration	NA	ext. mem.

**Table 5:** Wearable devices for simultaneous monitoring of environmental and behavioral domains (G2): part II.

Device	Sampling Rate (H)	Resolution/LoD/Coverage	Application	Data Usage
ART [68]	NA	O3: 10 ppb, VOC: 0.3 ppm to 30 ppm, O${}_{3}$: 10 ppb to 60 ppb	asthma management	cloud/server
MLMS-EMGN-5.1 [69]	motion tracking sensor: 50 Hz, CO and NO${}_{2}$: 0.2 Hz, UV index: 1 Hz, sound level: 2 Hz	NO${}_{2}$: 0.1 ppm, CO: 7.5 ppm, NO${}_{2}$: 0 ppm to 7 ppm, CO: 0 ppm to 1650 ppm	environmental	smartphone
[72]	NA	NA	environmental	smartphone
AMAS [73]	NA	NA	environmental for school age students	smartphone
[74]	1 Hz	NA	environmental	smartphone
UPAS [70]	NA	CH${}_{4}$: 0 ppm to 25 ppm, CO: 0 ppm to 30,000 ppm	toxic gases monitoring in oil and gas industries	PC
[75]	3 modes: 1 L\min & 2 L\min & 3 L\min air flow	10 to 25 g	environmental	smartphone
Eco-Mini [76]	NA	NA	environmental and activity	cloud/server
[71]	NA	NA	air quality	PC

### 3.3. Environmental, Behavioral, and Physiological Monitoring

G3 wearable devices measure 3 domains. We follow the same policy with G2 and give the concentration to the environmental and physiological domains to present the results. Temperature and humidity are measured by 9 and 7 devices, respectively. Air pollutants, including CO${}_{2}$, CO, and NO${}_{2}$, are measured by 4, 3, and 2 devices, respectively. The physical environmental parameters consist of UV, pressure, and sound level, as well as particle matter, VoCs, O${}_{3}$, and O${}_{2}$ are integrated, each in 1 device (Figure 4, right).

Extending the monitoring to the physiological domain shows that vital signals are the highest priority. Heart rate (HR), skin temperature (ST), and breathing rate (BR) each is measured by 8, 6, and 3 devices, respectively. Skin impedance (SI), Electroencephalography (EEG), Photoplethysmogram (PPG), and pulse oximetry (SpO${}_{2}$) sensors, are integrated, each in 1 device (Figure 4).

**Figure 7:**
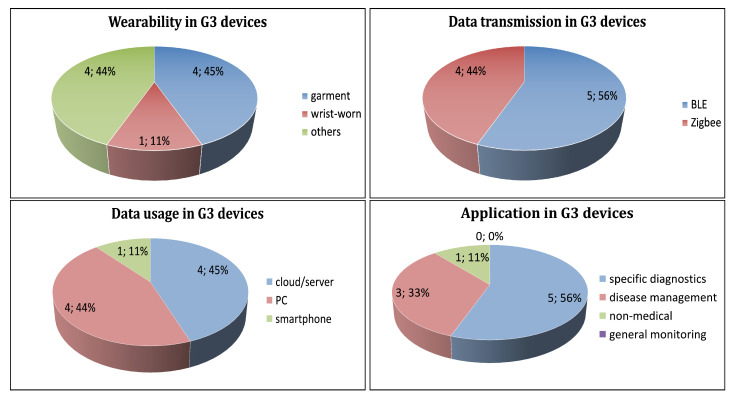
Distribution of G3 wearable devices according to: mode of wearability (**top-left**), data transmission (**top-right**), data usage (**bottom-left**), and application (**bottom-right**) of the wearable devices.

In G3, the number of domains is three, thus, it is expected the degree of convenience is reduced reflected in mode of wearability. Forty-five percent (4) of the devices are worn as the garment, 11% (1) of the devices are worn on wrist, and 44% (4) of the devices classified as others (distributed approaches and attached to users) (Figure 7, top-left).

BLE is the most used protocol, in 55% (5) of the devices, followed by Zigbee in 45% (4) of the devices. One device is not specified the protocol (Figure 7, top-right).

Forty-five percent (4) of the devices, transmit the data to a cloud/server. Forty-four percent (4) of the devices transmit the data to a PC, and 11% (1) of the devices transmit the data to a smartphone (Figure 7, bottom-left). Fifty-six (5), 33% (3), and 11% (1) of the devices are used for specific diagnostics, disease management, and non-medical, respectively (Figure 7, bottom-right).

Authors in Reference [77] have reported 133 € as the final cost of the prototype. We review the principles, features and specifications, and applications of G3 devices in Table 6 and Table 7.

**Table 6:** Wearable devices for simultaneous monitoring of environmental, behavioral, and physiological domains (G3): part I.

Device	Wearability	Parameters	Energy Consumption	Data Transmission
[77]	wrist-worn	CO, NO${}_{2}$, T, H, P, UV, SL, motion tracking, ST, HR	18.35 mA, 12.53 h	SR: BLE, ext. mem
[78]	2 parts: attachment	CO${}_{2}$, T, H, UV, ST, HR	NA	SR: BLE, LR: LoRa
[79]	garment	CO, T, posture, HR	NA	SR: Zigbee
[80]	2 parts: waist-worn and chest-worn	PM${}_{2.5,10}$, T, H, light, BR, acceleration	NA	SR: BLE, ext. mem.
[81]	3 parts:wrist-worn, chest-worn, and spiromerter	O${}_{3}$, VoC, motion, T, H, HR, BR, SI, PPG, ECG, motion	0.83 mW (wrist-worn)	SR: BLE
[82]	garment	CO${}_{2}$, T, HR, ST, acceleration	NA	SR: Zigbee
[83]	helmet	CO${}_{2}$,O${}_{2}$, T, H, P, HR, ST	0.5 A	SR: Zigbee
[84]	garment	NO${}_{2}$, T, H, HR, ST, movement	NA	SR: BAN wireless communication
[85]	garment, pair of boots	CO${}_{2}$, CO, T, H, BR, HR, ST, oxygen saturation, position, posture, motion	7 h	SR: Zigbee

**Table 7:** Wearable devices for simultaneous monitoring of environmental, behavioral, and physiological domains (G3): part II.

Device	Sampling Rate (H)	Resolution/LoD/Coverage	Application	Data Usage
[77]	CO and NO${}_{2}$: 1 Hz, SL: 2 Hz, UV index: 1 Hz, ST: 15 s time interval, PPG: 15 s time interval, motion sensors: 50 Hz	NO${}_{2}$:1 ppm, CO: 5 ppm, SL: 1 dB, UV: 1 index, ST: 1 °C, pressure: 1 hpa, temp.: 1 °C, H: 1% RH/ SL: 32 db to 85 dB, NO${}_{2}$: 0 ppm to 52.8 ppm, CO: 0 ppm to 1680 ppm, UV index: 0 to 9	COPD disease	smartphone
[78]	NA	NA	safety & health for industrial workers	cloud/server
[79]	NA	NA	firefighters	PC
[80]	NA	NA	asthma & COPD disease	cloud/server
[81]	VOC: 0.02 Hz, T & H: 10 Hz. PPG & accelerometer: 30 Hz. ECG:50 Hz	NA	respiratory disease	cloud/server
[82]	NA	NA	firefighters	cloud/server
[83]	NA	CO${}_{2}$:100 ppm to 600 ppm, O${}_{2}$: 18.5 to 22%, T: 10 °C to 40 °C, ST: 35 °C to 39 °C, P: 0.8 Bar to 1.2 Bar, H: 10% RH to 90% RH	work inharsh environments (mining)	PC
[84]	NA	NO${}_{2}$: 0.1 ppm/ NO${}_{2}$: 0 ppm to 10 ppm	firefighters	PC
[85]	NA	NA	firefighters	PC

## 4. Discussion

The four influencing domains are interwoven; thus, simultaneous monitoring is essential for comprehensive evaluation of the subject health. Air pollutants, including toxic, hazardous gases, and particular matter, influence the disease, such as cardiovascular, lung cancer, and respiratory. The risks of exposing to such pollutants have been already well identified and documented. New research has been conducted to evaluate the effect of air pollutants on the brain and mental illness (e.g., depression). The research shows the correlation between psychological status of subjects with air pollution. In the other words, higher level of air pollutants reduces the time that subject spent outdoor. Decreasing the physical activities outside consequently leads to worsening the psychological distress, including limited exposure to sunlight and social isolation [86,87,88,89].

### 4.1. Current Status of Wearables

Wearables are major means of personalized health monitoring for proactive healthcare [90,91,92]. The state of the art is concentrated on WBAN consisting several wearables interacting and transmitting data through the mesh/star topology to a gateway and eventually to a cloud/server for data fusion, analysis, and decision-making [93,94]. However, several limitations still exist, including wearability [95], power consumption [96], data accuracy [97], frequent calibration [98], restriction of data transmission [99], user privacy [100], and data security [95].

Due to importance of environmental domain, and its interactions with the other domains [101], we have categorized current wearable sensor systems into the groups G1 to G4, all including the environmental domain. Thus, it is expected to have the highest number of parameters from the environmental domain in the all groups. However, more often the wearable devices have been only used in short periods, e.g., a clinical trial, as they suffer from reliability, accuracy, and cannot support sufficient effective data on particular diseases [102].

#### 4.1.1. Application in Reactive and Proactive Healthcare

Ludwig et al. categorize the application of wearables into reactive healthcare (handling adverse conditions and events) and proactive healthcare (assessing the state of health) [103]. Automated fall or cardiac emergency detection are reactive healthcare and may lead to emergency calls. Monitoring known disease, for instance, are proactive healthcare. Therefore, simultaneous monitoring of environmental (e.g., air quality), behavioral (e.g., physical activity), and physiological parameters (e.g., heart rate, skin temperature) in combination the parameters from the psychological domain (e.g., emotion) can proactively assess the status (or status changes) of user health to predict and alert “long before occurrence” (principle 1,2: complete and continuous) [15]. However, the user must decide to use wearables daily and recharge and interconnect them as required by the devices. Costs and Convenience widely impact the acceptance of wearables [104,105,106].

**Figure 8:**
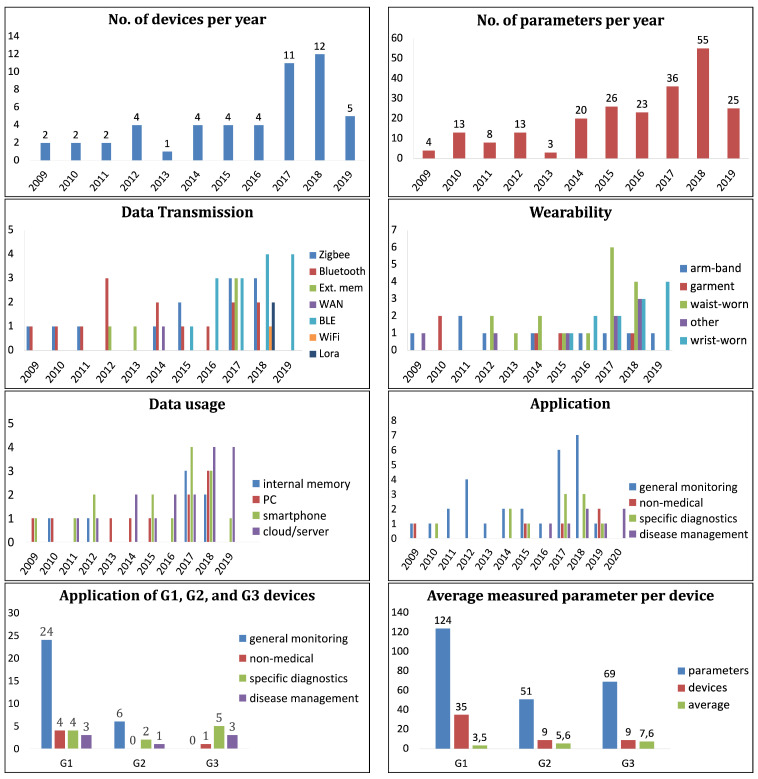
A statistic analysis of the wearables between 2009 and 2019 in the aspects of production and parameters (**top line view**), data transmission and wearability (**second line view**), data usage and application (**third line view**), and application of all groups and average parameters measurement (**bottom line view**).

**Figure 9:**
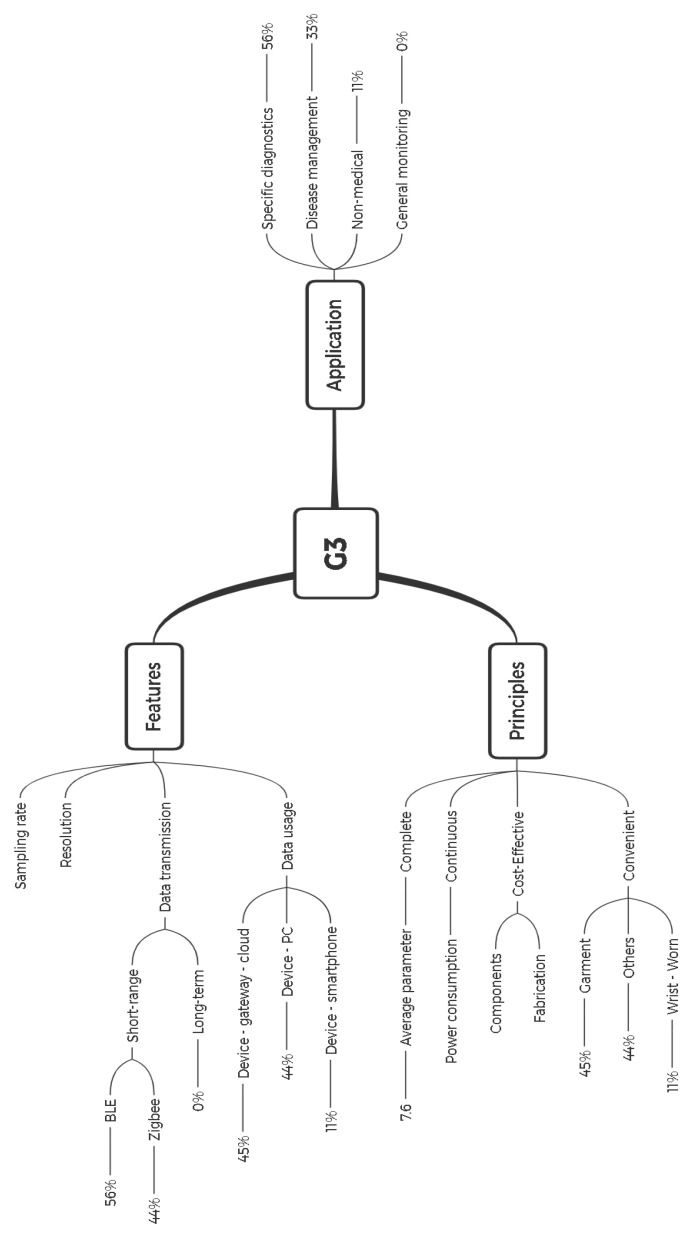
G3 analysis in terms of features, principles, and application. In G3, no device has been designed supporting general monitoring.

Tracing the wearables between 2009 and 2020 shows the significant growth of devices in the area of specific application and disease management rather than general applications. In the same manner, investigation on the general application of devices in G1, G2, and G3 indicate reducing the devices from 24 to 6 and eventually zero in G3 devices. Similarly, disease and specific application devices have grown their share from 3 in G2 to 8 in G3 devices (Figure 8, bottom line view, left). In particular, References [77,80,81] have been designed for monitoring COPD, asthma, and respiratory disease, respectively. Besides, References [79,82,84,85] have been designed for firefighters which are applied in health and safety (H&S). Considering the number of devices and applications, G3 is the most disease and specific application. Having said that, we studied the most influencing factors in structuring G3, resulting in greater correlated diverse heterogeneous data, BLE data transmission, garment and wrist-worn monitoring, and device → gateway → cloud. This supports acceptance of growing role of wearables in proactive healthcare. We have visualized the impact of different factors on devices to construct G3 and their application (Figure 9).

Our study shows the number of devices ascending from 2 to 12 between 2009 and 2018 (Figure 8, top-left). This supports the statement that our healthcare systems currently transits from treatment after diagnosis towards prediction, prevention, and proactive personalized healthcare [107]. Enhancing the penetration rate of wearables in real studies requires user and physician satisfactions [22]. Thus, as a matter of fact, improving continuous monitoring is impacted by reducing the power consumption, which is consequently function of mode of data transmission. The statistics between 2009 and 2019 indicate the increasing share of BLE in data transmission (Figure 8, second line, left). Besides, wrist-worn devices, as the most convenient mode of wearability have jumped to the top in 2019 (Figure 8, second line, right). Weight of the wearables as an important factor has been relatively reduced such that the wearables have been improved from 2011 to 2019 by reducing the weight from 251 g to 52 g, approximately.

Although information on the costs of the prototypes is insufficient, the general range is about 140 US$. Such a range may fit to user’s expectations and significantly motivates contributors to turn prototypes into products.Nevertheless, wearables are must contribute in to decision-making. This is accomplished by automated data fusion of multiple sensors from different domains. In general, the transition of wearables from G1 to G3 is accompanied with increasing average number of measurement from 3.5 to 7.6 parameters per device (Figure 8, bottom-right). The total number of parameters from 2009 to 2018 has grown from 4 to 56 (Figure 8, top-right), showing that wearable devices increase their role in proactive healthcare by extending their domain and range of monitoring.

#### 4.1.2. Big Data and Integration of Wearables into Healthcare Systems

Bid data is often described by four Vs, volume (feature: sampling rate and power consumption), velocity (feature: data transmission), variety (principle: complete), and veracity (feature: resolution and LoD). Wearables have the capability and potential to generate big data. The battery power is the most limiting factor of data volume and complexity. A trade-off between sampling and transmission rates is required to restrict the power consumption [108]. However, not all developers sufficient information (e.g., experimental data on battery volume or current leakage) on the operating time of the devices. Thus, we cannot conclude quantitatively but rather qualitatively: As the number of monitoring domains and parameters have increased, the sampling rate, rate of data transmission, and/or wearability have been optimized, depending on the particular application.

In addition, care delivery, supporting real-time physician’s feedback, and telemedicine requires bidirectional communication. Therefore, data usage (propagation) potentially impacts and drives wearables towards integration and application [109]. Their potential of generating big data opens wearables to external healthcare systems, such as hospitals or rescue teams. The data transmission from devices → personalized gateway → cloud/server and from a device → smartphone have reached from 1 to 3 and from 0 to 4 in 2018 and 2019, respectively (Figure 8, third line, left). Personalized gateways are embedded in the short and long terms [110]. We expect further increase of smartphone as personalized gateway in telemedicine and penetration in remote regions. Additionally, moving toward 5G network further highlight the future role of smartphone as personalized gateways improving the efficiency of data transmission by reducing the data loss and faster delivery [111].

### 4.2. Future Group of Wearables (G4)

Although, the transition from distributed wearable systems to integrated centralized wearable systems is traced from G1 to G3, the devices are still lacking complete monitoring of healthcare and well-being, which required complete monitoring of all four WHO domains. Therefore, the psychological domain needs particular attention in future wearables [112,113].

**Figure 10:**
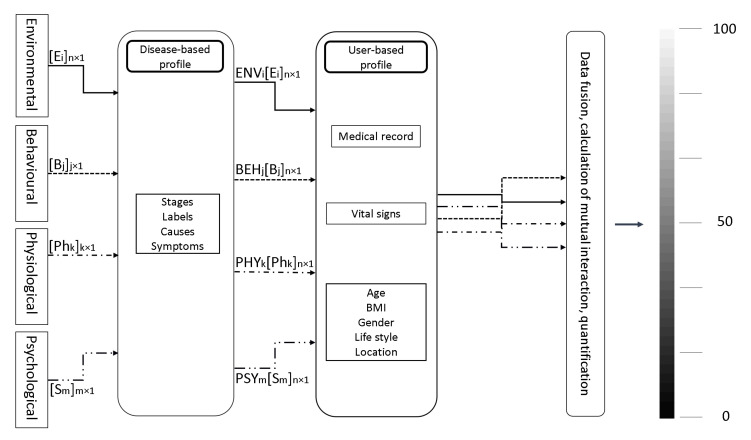
Customized and quantified output for future wearables. BMI stands for body mass index. E, B, Ph, and S stand for environmental, behavioral, physiological and psychological, each with number of parameters from 1 to n, j, k, and m, respectively. ENV, BEH, PHY, and PSY are the coefficient of environmental, behavioral, physiological, and psychological domains, weighting the parameters.

However, simply delivering raw data to physicians without meaningful interpretation does not accelerate decision-making [114]. Hence, the fourth group of wearables (G4) further demands a customized and quantified output with respect to disease and user’s health status. This personalization enables a meaningful contribution to the decision-making of physicians [22]. Following this approach, we propose simultaneous monitoring of all four influencing domains, followed by a two-layer filter and computations of parameters’ interactions (Figure 10). In the first step, parameters from all four domains are considered. We suggest to measure and quantify the output with respect to the target disease and the customized profile of the patient/user. The first filter is disease profile. It includes numbers of disease profiles and is uniquely proposed to be structured according to each disease [115]. Each disease profile must include the factors describing the disease comprehensively. Comparison of the disease profile with the incoming measured parameters that the user is exposed to, leads to filtering the non-effective parameters, alleviate the less effective parameters, and gives higher weights to effective parameters. Therefore, the number of parameters might be changed and the influencing coefficient of ENV${}_{i}$, BEH${}_{j}$Please define if appropriate. , PHY${}_{k}$, and PSY${}_{m}$ are added to each parameter.

The second filter highlights parameters as effective according to the user’s profile [116]. All important information such as the anamnesis are recorded in this profile. The vital signs of the user are monitored for a limited period of time to identify drifts out of the user’s normal range. We expect that the incoming parameters are treated differently with respect to physiological status of the user.

In the last step, the most impacting parameters are correlated. The impact of each parameter is calculated within and across the four domains. The output is dynamic for a particular disease and user. It covers five ranges: regular (healthy), abnormal, pathological, crucial (warning), and exceptional (emergency). It is quantified from 1 to 100 indicating the risk of emergency. The output value significantly indicates to both users and physicians the necessary steps to take in prevention and treatment.

## 5. Conclusions

In conclusion, simultaneous monitoring of the environmental, behavioral, physiological, and psychological domains is desirable due to mutual interactions among the domains and their parameters. We have considered the environmental domain as a central player, which independently influences the other domains. According to these interwoven domains, we have categorized wearables into groups (G1 to G4). Existing wearables show a significant contribution of G3 devices for disease management (e.g., COPD and asthma) and, therefore, proactive healthcare. Increasing the penetration rate of smartphone as personalized gateway supports the integration of wearables into our healthcare systems for real-time assessment of user’s health and immediate care delivery. We propose future wearables to focus on integration of parameters from the psychological domain.

## Figures and Tables

**Table 1:** Comparison of different wireless communication protocols used in wireless body area networks (WBANs). P2P and LPWAN stand for Peer to Peer and Low Powered Wide-Area Network, respectively.

Wireless Technology	Protocol	Frequency Bands	Data Rate	Data Range	Network Topology	Power Consumption
Bluetooth (classic)	IEEE 802.15.1	2.4 GHz	1–3 Mb/s	∼100 m	P2P, Star	Low
BLE	IEEE 802.15.1	2.4 GHz	1 Mb/s	∼100 m	P2P, Star	Very Low
ZigBee	IEEE 802.15.4	868,915 (MHz), 2.4 (GHz)	250 Kb/s	∼10–100 m	P2P, Star, Tree, Mesh	Medium
LoRa	LPWAN	868,915 MHz	50 Kb/s	∼10 Km	Star, Mesh	Very Low
WiFi	IEEE 802.11	2.4, 3.7, 5 GHz	>45 Mb/s	∼30–250 m	P2P, Star, Tree	High

## Data Availability

Not applicable.
